# Determination of language areas in patients with epilepsy using the super-selective Wada test^☆^

**DOI:** 10.1016/j.ibneur.2022.08.002

**Published:** 2022-08-10

**Authors:** Kazuo Kakinuma, Shin-ichiro Osawa, Hiroaki Hosokawa, Marie Oyafuso, Shoko Ota, Erena Kobayashi, Nobuko Kawakami, Kazushi Ukishiro, Kazutaka Jin, Makoto Ishida, Takafumi Sato, Mika Sakamoto, Kuniyasu Niizuma, Teiji Tominaga, Nobukazu Nakasato, Kyoko Suzuki

**Affiliations:** a Department of Behavioral Neurology and Cognitive Neuroscience, Tohoku University Graduate School of Medicine, 2-1 Seiryo, Aoba, Sendai, Miyagi 980-8575, Japan; b Department of Neurosurgery, Tohoku University Graduate School of Medicine, 2-1 Seiryo, Aoba, Sendai, Miyagi 980-8575, Japan; c Department of Rehabilitation, National Hospital Organization Sendai-Nishitaga Hospital, 2-11-11 Kagitorihoncho, Taihaku, Sendai, Miyagi 982-8555, Japan; d Department of Neurology and Stroke Medicine, Yokohama City University School of Medicine, Graduate School of Medicine, 3-9 Fukuura, Kanazawa, Yokohama, Kanagawa 236-0004, Japan; e Department of Epileptology, Tohoku University Graduate School of Medicine, 2-1 Seiryo, Aoba, Sendai, Miyagi 980-8575, Japan; f Clinical Physiological Laboratory, Tohoku University Hospital, 1-1 Seiryo, Aoba, Sendai, Miyagi 980-8574, Japan; g Department of Neurosurgical Engineering and Translational Neuroscience, Graduate School of Biomedical Engineering, Tohoku University, 2-1 Seiryo, Aoba, Sendai, Miyagi 980-8575, Japan; h Department of Neurosurgical Engineering and Translational Neuroscience, Tohoku University Graduate School of Medicine, 2-1 Seiryo, Aoba, Sendai, Miyagi 980-8575, Japan

**Keywords:** ssWada, super-selective Wada test, fMRI, functional magnetic resonance imaging, MEG, magnetoencephalography, MCA, middle cerebral artery, M2 sup, M2 superior division of middle cerebral artery, M2 inf, M2 inferior division of middle cerebral artery, EEG, electroencephalography, ECD, equivalent current dipole, CTA, computed tomography angiography, ECS, electrocortical stimulation, Epilepsy, Wada test, Lateralization, Epilepsy surgery, Functional mapping, Preoperative planning

## Abstract

The Wada test is the gold standard for determining language-dominant hemisphere. However, the precise determination of language areas in each patient requires more invasive methods, such as electrocortical stimulation. Some studies have reported the use of anesthetic injection into selective cerebral arteries to predict postoperative function. To assess the function of the anterior and posterior language areas separately, we developed an advanced test named the “super-selective Wada test” (ssWada). The ssWada procedure is as follows: an endovascular neurosurgeon identifies the arterial branches of the middle cerebral artery (MCA) perfusing the anterior language area of the inferior frontal gyrus and the posterior language area of the posterior part of the superior temporal gyrus using angiography. Behavioral neurologists assess language symptoms before and after propofol administration using a microcatheter tip in the selected arterial branch. From 30 serial patients with epilepsy who underwent ssWada test at Tohoku University Hospital, we retrospectively reviewed patients in whom multiple areas in the bilateral MCA region was examined. Eight cases were identified in this study. All eight cases had been considered for resection of the area overlapping the classical language area. Three of the eight cases were left-dominant, and the within-hemisphere distribution was also considered typical. One case was determined to be left-dominant but atypical in the intra-hemispheric functional distribution. Two cases were right-dominant, and the intra-hemispheric functional distribution was considered a mirror image of the typical pattern. The remaining two cases were considered atypical, not only in terms of bilateral language function, but also in terms of anterior-posterior functional distribution. This case series demonstrates the potential utility of ssWada in revealing separate function of the anterior and posterior language areas. The ssWada allows simulation of local surgical brain resection and detailed investigation of language function, which potentially contributes to planning the resection area. Although indications for ssWada are quite limited, it could play a complementary role to noninvasive testing because it provides information related to resection using a different approach.

## Highlights

• The super-selective Wada test (ssWada) injects anesthetic locally into the brain.
• The ssWada simulates states of post-resection functional impairment.
• The ssWada successfully localized different components of language separately.

## 1 Introduction

Focal resection is a potential treatment option for refractory epilepsy. However, neurosurgical operations involving brain resection carry the risk of cognitive impairment, especially postoperative aphasia. As a preoperative assessment for brain surgery, localization of the language area is a critical issue (Hamberger, 2007), particularly in patients with epilepsy because they have a higher incidence of atypical language distribution (Powell and Duncan, 2005, Berl et al., 2014). In neurosurgery, the gold standard for determining language areas is electrocortical stimulation (ECS) mapping, which can transiently and selectively inhibit brain function in a small region using a temporary current (Hamberger, 2007). However, this technique requires highly invasive neurosurgical procedures including craniotomy or intracranial electrode placement. It may also produce a false-negative result, possibly due to diffuse or atypical localization of brain function and insufficient strength and coverage of electrical stimulation. Functional magnetic resonance imaging (fMRI) and magnetoencephalography (MEG) are currently used for noninvasive determination of language lateralization. These noninvasive tests have historically shown insufficient accuracy, but recent advances in both equipment and methodology have led to gradual improvement in accuracy (Bauer et al., 2014, Massot-Tarrús et al., 2017, Papanicolaou et al., 2018, Qadri et al., 2021). While noninvasive tests generally coincide with invasive tests, it is sometimes suggested that noninvasive tests are weighted more toward sensitivity than specificity (Rolinski et al., 2019). In other words, a brain region exhibiting task-related activation does not necessarily produce corresponding cognitive symptoms when it is resected. Kemp et al (Kemp et al., 2018). pointed out the risks of depending solely on noninvasive tests and concluded that the complementary use of invasive and noninvasive tests is beneficial.

The intracarotid anesthetic (Wada) test is a standard tool for preoperative language assessment that determines language lateralization (Wada and Rasmussen, 1960). This shows a high concordance with the ECS (Wyllie et al., 1990). In the Wada test, a short-acting anesthetic is injected through the left or right internal carotid artery to temporarily suppress the function of the ipsilateral cerebral hemisphere. It is classically performed with amobarbital; however, in recent years, it has been performed with propofol because of its availability (Curot et al., 2014, McCleary et al., 2018). In recent years, the Wada test has been used to predict the postoperative memory performance. While the availability of the memory Wada test has been supported by several reviews, the superiority of the classical Wada test over noninvasive tests has been questioned owing to various limitations (Qadri et al., 2021, Conradi et al., 2021, Massot-Tarrús et al., 2020). The Wada test can only determine language lateralization and provides no information about within-hemisphere distribution. It also has some limitations in determining the laterality. For example, bilateral anesthetic delivery through the circle of Willis blurs laterality determination in some patients. In addition, intracarotid propofol injection had adverse effect in 33% of patients (Mikuni et al., 2005). Variations in the Wada test have existed in the past, in which anesthetics were injected into the posterior cerebral artery (Jack et al., 1989) or the middle cerebral artery (MCA) (Fujii et al., 2011). The selective MCA Wada test developed by Fujii et al (Fujii et al., 2011). can efficiently assess language function and reduce complications related to intracarotid propofol injection. In parallel to this invention, some papers have reported cases in which selective anesthetic infusion into the cerebral artery branch contributed to the prediction of postoperative complications (Hajek et al., 1998, Urbach et al., 2002, Yamashita et al., 2021).

For detailed preoperative evaluation of eloquent brain functions, we refined the protocol by propofol injection into a branch of the MCA, named the “super-selective Wada test” (ssWada), which involves simulation of regional resection and systematic language evaluation. This method can evaluate the motor and sensory language areas separately. In addition, it minimizes the previously pointed limitations of the Wada test, such as conscious disturbances. This study aimed to conclude the clinical question of whether the application of the ssWada enables to describe the additional information about the relationship between detailed language function and brain areas perfused by the branches of the MCA. Here, we present eight cases and their inter- and intra-hemispheric language distributions.

## 2 Material and methods

### 2.1 Patients

Patients with intractable epilepsy who underwent the ssWada test between January 2018 and November 2021 at Tohoku University Hospital were retrospectively examined (Supplementary Table 1). From a series of 30 patients, we extracted eight who underwent ssWada in the M2 superior division (M2 sup) and M2 inferior division (M2 inf) of the MCA on the language-dominant side, as well as at least one MCA branch (M2 sup, M2 inf, of M1) on the language non-dominant side. In all patients, the seizure focus was preoperatively evaluated using a combination of imaging findings, ictal electroencephalography (EEG), and long-term video EEG. Table 1 presents the patient demographics.

**Table 1:** Patient demographics.

	Age	Sex	Handedness	Onset age	EEG abnormality	MRI	PET	Resection Area	Etiology
Case 1	38	M	Rt	3	Blt O, m-pT, P	Negative	Rt O area	Rt O	FCD Type IIa
Case 2	18	M	Lt	4	Lt O, m-pT, P	Blurring GW junction at O	Lt O, T area	Lt O	FCD Type IIb
Case 3	49	M	Rt	4	Lt T	Negative	Lt T, O area	Lt T	FCD Type Ia
Case 4	29	F	Rt	24	Rt T, P	Negative	Rt STG, insula	Rt Insula	Encephalitis
Case 5	38	M	Lt	15	Lt a-mT	Tumor at Lt MTG	Lt T area	Lt T	DNT
Case 6	46	M	Rt	3	Rt T	Negative	Lt T area	Rt T	FCD Type IIb
Case 7	29	M	Rt	23	Lt T, P	Cortical thickness at Lt STG, SMG and AG	Negative	Lt T	FCD Type IIb
Case 8	32	F	Rt	1	Lt a-mT, F,	Cortical thickness at lt. OFA, SFG, MFG, IFG, PreCG and PostCG	Negative	Lt F	mMCD

M, male; F, female; Rt, right; Lt, left; Blt, bilateral; F, frontal; T, temporal; P, parietal; O, occipital; a, anterior; m, middle; p, posterior; GW, gray matter-white matter; MTG, middle temporal gyrus; STG, superior temporal gyrus; OFA, orbitofrontal area; SFG, superior frontal gyrus; MFG, middle frontal gyrus; IFG, inferior frontal gyrus; PreCG, precentral gyrus; PostCG, postcentral gyrus; FCD, focal cortical dysplasia; mMCD, mild malformations of cortical development; DNT, dysembryoplastic neuroepithelial tumor.

### 2.2 fMRI and MEG for language dominance

Simple verb-generation tasks with visual and auditory presentations were used in combination with fMRI to determine language dominance. Language lateralization was determined by visual inspection of the activated areas by a neuroradiologist who was blinded to the clinical data. In addition, language mapping was performed using MEG in conjunction with an auditory word recognition task (Papanicolaou et al., 2004).

### 2.3 The ssWada

#### 2.3.1 Endovascular procedure

All the procedures were performed by endovascular neurosurgeons in an endovascular neurosurgical suite. After diagnostic cerebral angiography, we introduced a 6-Fr guiding catheter into the cervical segment of the internal carotid artery proximal to the targeted area. Next, super-selective catheterization was performed to lead the microcatheter tip to the intracranial artery.

After the perfusion area was confirmed by super-selective angiography using a microcatheter, 1 mg/mL propofol was infused at a rate of 1 mL/s after the baseline evaluation of the neurological findings. The amount of infused propofol was determined based on a previous report (Takayama et al., 2004) and the diameter of the target vessel on angiography. We used 7.5–10 mg or 5–7.5 mg, when the selected artery was the M1 segment or M2 segment (superior and inferior MCA trunks in many of the included cases), respectively. Scalp EEG was monitored to confirm electrophysiological changes during drug infusion.

#### 2.3.2 Determination of target vessel

The infusion site and order of the tested vessels were determined to obtain the essential information required to delineate the functional border for the expected cortical resection area and to minimize the functional deterioration risk in advance of craniotomy. We antecedently analyzed intracranial vascular anatomy using computed tomography angiography (CTA) to determine the optimal order for testing vessels in relation to the previously reported functional anatomy of the cortex. To evaluate the functionality and reserve capacity of the brain’s suspected epileptogenic zone in preparation for resection, we selected the vessels as follows. First, we chose the vessel that perfuses the largest area responsible for the suggested postoperative neurological deficit. Second, we selected the major branch adjacent to the previously tested vessel in the ipsilateral hemisphere. Third, we moved the guiding catheter to the contralateral side and selected the vessel perfusing the area homologous to that of the affected side. For example, in cases of suspected epileptic foci in the left frontal lobe, we first selected the M2 sup of the left MCA, perfusing the anterior language area of the inferior frontal gyrus. We then chose the left M2 inf perfusing the posterior language area of the posterior part of the superior temporal gyrus. Third, we chose the distal part of the M1 MCA segment on the contralateral side, perfusing the homologous area tested on the ipsilateral side. This was mainly due to the need to check for neurological consistency between contralateral and ipsilateral deficits.

#### 2.3.3 Neurocognitive tasks

Cognitive tasks were performed just before and after propofol administration: (A) As soon as the neurosurgeon selected the vessel and guided the catheter to the target branch, behavioral neurologists began the baseline cognitive assessment (tasks 1–8). (B) Immediately after the baseline cognitive assessment, the neurosurgeon injected propofol while the patient performed sequential speech (task 1). The effect of propofol was monitored using EEG and neurological signs (motor, visual, sensory, or language disturbance). (C) If EEG changes or any neurological signs were observed, we moved on to subsequent tasks (tasks 2–8). If both EEG and neurological changes were unclear, we checked the performance on task 1 for 20–30 s and then proceeded to the subsequent tasks (2–8). All procedures were performed consecutively without an interval of > 1 min.

The neurocognitive assessment included the following 8 tasks:

1. Sequential speech: counting or speaking sequential words, such as the day of the week.
2. Listening comprehension: following two simple verbal commands.
3. Repetition: repeating a word and a sentence.
4. Reading aloud (sentence): reading a sentence aloud.
5. Reading comprehension: following a written instruction.
6. Reading aloud (words): reading four kanji (morphogram) words and four kana (phonogram) words.
7. Naming: naming eight object pictures.
8. Writing: dictating eight words (four kanji and four kana).

The stimuli were presented on an iPad placed approximately 30 cm from the patient’s eyes.

#### 2.3.4 Evaluating perfusion areas

**Fig. 1:**
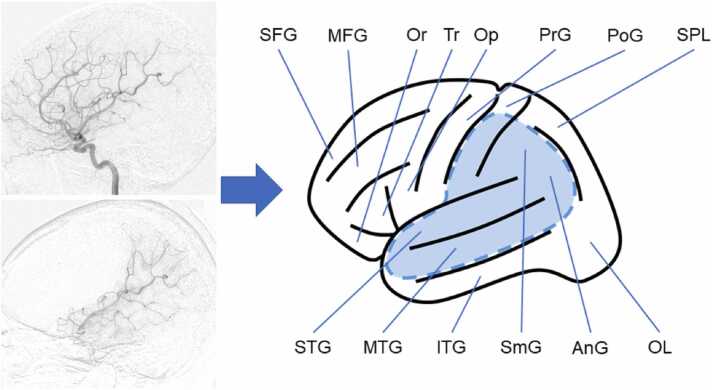
An example of plotting the perfusion area from the angiography results. The perfusion areas were estimated based on cerebral angiography by tracer injection into the cervical segment of the internal cerebral artery (top left) and super-selective injection into the M2 inferior division of the middle cerebral artery (bottom left). The perfused area is shown in color in the schema (right). AnG, angular gyrus; IFG, inferior frontal gyrus; ITG, inferior temporal gyrus; MFG, middle frontal gyrus; MTG, middle temporal gyrus; OL, occipital lobe; Op, opercular part of the IFG; Or, orbital part of the IFG; PoG, postcentral gyrus; PrG, precentral gyrus; SFG, superior frontal gyrus; SmG, supramarginal gyrus; SPL, superior parietal lobule; STG, superior temporal gyrus; Tr, triangular part of the IFG.

Based on cerebral angiography (CTA and MRI), we determined the artery perfusing the target cerebral region by visually inspecting and mapping it onto the schema shown in Fig. 1.

#### 2.3.5 Types of language dysfunction observed during propofol injection

Based on the performance of neurocognitive tasks, we classified propofol-induced language impairment into four categories:

1. Motor aphasia: Severe speech impairment with preserved comprehension.
2. Sensory aphasia: Fluent speech but other language deficits present.
3. Global aphasia: Impairment of both speech and comprehension.
4. No language impairment: Good performance on all language tasks.

## 3 Results

In all cases, the test was performed safely and no irreversible side effects were observed. All transient symptoms caused by propofol disappeared within 15 min of injection. The duration of EEG changes and neurological symptoms are described in detail in Supplementary Table 1**.** All cognitive assessments were performed within this period of time. No permanent neurological deficits were observed in any of the patients.

**Fig. 2:**
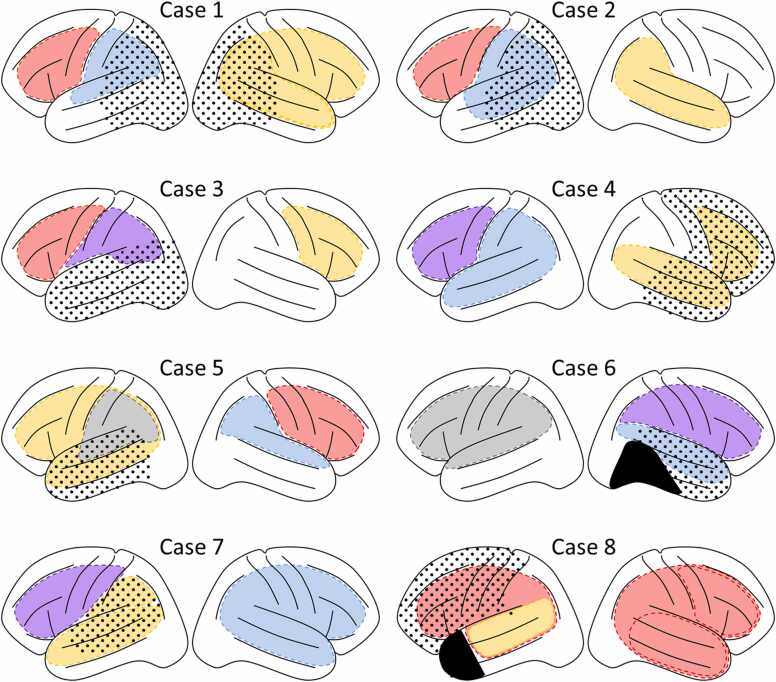
Perfusion areas of each arterial branch in eight patients. Each color corresponds to a symptom observed during propofol infusion as follows: red, motor aphasia; blue, sensory aphasia; purple, global aphasia; yellow, no language impairment; and gray, indeterminable. The posterior part of the right basal temporal lobe in case 6 and the left anterior temporal lobe in case 8 were resected by a previous operation (shown in black). Although the left temporoparietal region in Case 2 and the right hemisphere in Case 7 are shown in blue, the impairment in comprehension was mild, but anomia and word errors were prominent. The dots indicate areas that were considered for possible inclusion in the resection area prior to invasive examination.

Table 2 shows the language lateralization determined using fMRI, MEG, ssWada, and ECS. For cases in which we could determine the language area by ECS, the ECS-determined language laterality was consistent with the prediction of the ssWada in each case. Fig. 2 shows the distribution of cerebral vascular perfusion and induced language symptoms, and the maximum extension of the potential resection area estimated prior to invasive examination. The possible resection areas in these eight cases overlapped with the peri-Sylvian language areas. Therefore, the extent of these language areas in each patient must be carefully examined. Supplementary Table 2 summarizes the detailed language symptoms induced by the propofol injection. Four of the eight cases showed clear language impairment upon anesthetic injection in the left MCA branch and no language impairment in the right MCA branch, indicating left language dominance (Cases 1–4). Cases 5 and 6 showed right language dominance. Cases 7 and 8 showed language symptoms after propofol injection in both the left and right MCAs, although the language-predominant hemisphere was assumed to be left and right, respectively.

**Table 2:** Language lateralization determined by different tests.

	fMRI	MEG	ssWada	ECS
Case 1	Indeterminable	Bilateral	Left	Indeterminable*
Case 2	Right>Left	Right	Left	Left
Case 3	Left	Bilateral	Left	NE
Case 4	Left	Left	Left	NE
Case 5	Right>Left	Left	Right	not Left
Case 6	Indeterminable	NE	Right	Right
Case 7	Indeterminable	Left	Left	Left
Case 8	NE	Bilateral	Bilateral	Right

fMRI, functional magnetic resonance imaging; MEG, magnetoencephalography; ssWada, super-selective Wada test; ECS, electrocortical stimulation; Right>Left, relatively right; NE, not examined; * insufficient electrode coverage in potential language areas.

### 3.1 Case 1

A 38-year-old right-handed man developed epileptic seizures at 3 years of age. He developed a visual aura in the right upper visual field, followed by impaired awareness, which occasionally evolved into generalized tonic-clonic seizures. The seizures were drug-resistant, and the patient was admitted with a complaint of seizures occurring several times a week. Magnetic resonance imaging (MRI) revealed focal cortical dysplasia in the right occipital lobe. Long-term video EEG monitoring revealed interictal right temporo-occipital spikes. Perimeter examination revealed left upper quadrant anopsia.

The patient showed motor and sensory aphasia after anesthetic injection into the M2 sup and M2 inf on the left side. No language deficits were induced by injection into the right MCA. We determined that this patient had typical language lateralization, and that resection of the right temporo-occipital focus could be performed without concern for alexia or agraphia.

After ssWada, intracranial electrodes were placed on the bilateral occipital lobes and parts of the temporal lobes. As ssWada indicated the posterior language area on the left side, the electrodes were placed only in a small part of the right temporal lobe. Thus, the posterior language area on the left side was not determined using ECS mapping. No language impairment was observed after resection of the right occipital focus.

### 3.2 Case 2

An 18-year-old left-handed man developed focal impaired awareness seizures at 4 years of age. The seizures were drug-resistant, occurred multiple times every week, and evolved into generalized tonic-clonic seizures several times a year. Long-term video-EEG monitoring revealed continuous interictal spikes in the left parieto-occipital region.

Although fMRI and MEG indicated right language dominance, ssWada showed a typical language distribution in the left hemisphere. Propofol injection into the left M2 sup and left M2 inf caused motor and sensory aphasia, respectively. Injection into the right MCA branch did not induce language deficits, although visuospatial impairment prevented reading.

Based on the results of ssWada, intracranial electrodes were placed on relatively large areas of the left hemisphere, including the left occipitotemporal and parietal lobes. The ECS mapping of the left temporal lobe inhibited naming and verbal comprehension, thereby confirming the posterior language area. Resection of the epileptic focus, centered on the left occipital lobe, was performed with great care to avoid the language area. No language impairments were observed postoperatively.

### 3.3 Case 3

A 49-year-old right-handed man developed focal impaired awareness seizures at 4 years of age. Despite treatment with multiple antiepileptic drugs, the patient experienced seizures daily. Long-term video EEG monitoring revealed interictal left temporo-occipital spikes. It also showed that his habitual seizures were accompanied by fast wave activity in the same region. The language-dominant hemisphere determined by fMRI did not match that determined by MEG.

On ssWada, anesthetic injection into the left M2 inf induced impairments in both spontaneous speech and listening comprehension, since it perfused some ventral portions of the precentral and premotor cortices as well as the temporoparietal area. Anesthetic injection into the left M2 sup induced motor aphasia. Anesthetic injection into the right MCA did not cause any language symptoms. These findings indicate that the language-dominant hemisphere was on the left, and the distribution of language areas was typical.

Following ssWada, intracranial electrodes were placed on the left temporo-occipital areas; however, the occurrence of intracranial hypertension during the course of the procedure led to the cancellation of ECS mapping. Focal resection involving the left temporal lobe resulted in language impairment.

### 3.4 Case 4

A 29-year-old right-handed woman presented with a history of limbic encephalitis that started at the age of 21 and was successfully treated. She developed a visual aura of familiar scenes followed by impaired awareness at 24 years of age. Despite intensive medical treatment with multiple drugs, the seizures occurred several times a day. Long-term video EEG monitoring revealed focal sensory seizures arising from the right frontotemporal region. Brain MRI revealed lesions in the bilateral hippocampus and insular cortex. She showed amnesia, as indicated by a delayed memory index of 71 on the Wechsler Memory Scale-Revised. However, her general cognitive functions were well preserved. The verbal and performance intelligence quotients of the Wechsler Adult Intelligence Scale-third edition were 101 and 129, respectively.

Because lesions were bilateral and the epileptic focus was relatively extensive, it was considered necessary to determine the language-dominant side and distribution of language areas using the ssWada and to predict postoperative functional deficits. On ssWada, the patient showed atypical language distribution in the left hemisphere, and global aphasia was observed upon anesthetic injection into the left M2 sup. Anesthetic injection into the left M2 inf induced sensory aphasia, whereas injection into the right MCA did not cause language deficits.

Following ssWada, intracranial electrodes were placed on the right frontotemporal areas, and intracranial EEG revealed that the ictal onsets were in the right insular cortex. Based on the results of the ssWada, language mapping using ECS was not performed in this patient. Postoperatively, no language impairments were observed.

### 3.5 Case 5

A 38-year-old left-handed man developed epilepsy at 15 years of age. He presented with an aura of déjà vu, dystonic posture of the right upper limb, and impaired awareness. Brain MRI showed a tumorous lesion in the left temporal lobe with low intensity on T1-weighted images and high intensity on T2-weighted and fluid-attenuated inversion recovery images. These findings suggested a dysembryoplastic neuroepithelial tumor. Furthermore, ^18^F-fluorodeoxyglucose positron emission tomography revealed hypometabolism in this area. Long-term video-EEG monitoring revealed interictal left anterior temporal spikes and ictal findings in the left temporal region, suggesting that the tumor was an epileptogenic focus. Noninvasive tests for determining language dominance yielded inconsistent findings; specifically, right and left dominance were observed on fMRI and MEG, respectively.

ssWada showed right language dominance. Anesthetic injection into the right M2 sup induced motor language impairment and slight comprehension difficulty, whereas injection into the right M2 inf caused sensory aphasia. Furthermore, anesthetic injection into the left M2 inf resulted in transient confused responses to verbal commands. This was considered a nonverbal symptom given the absence of language deficits upon injection into the left proximal MCA. Subsequent ECS mapping using intracranial electrodes on the left temporal and parietal lobes revealed no obvious language areas in these regions. After resection of the left temporal lesion, the patient’s language function was well preserved.

### 3.6 Case 6

A 46-year-old right-handed man developed focal impaired awareness seizures at 3 years of age. Since drug treatment did not control his weekly seizures, he underwent intracranial electrode placement and focal resection of the right basal temporal region at the age of 27. A conventional Wada test performed prior to the first surgery indicated that his language function was lateralized to the right hemisphere. Intracranial electrodes detected spiny waves in a relatively deep region of the posterior part of the right inferior temporal gyrus, where computed tomography showed cortical dysplasia. Despite the initial surgery, seizure remission was not achieved. Subsequently, the patient was referred to our hospital to determine the indication for further treatment with a second surgery.

ssWada showed right language dominance. The injection into the right M2 inf caused sensory aphasia. Injection into the right M2 sup, which extended more posteriorly than normal and perfused a wide portion of the right hemisphere, induced global aphasia. Similar to the trial in the left M2 inf in case 5, the anesthetic injection into the left M1 of case 6 resulted in an acute confusional state, followed by good speech and language comprehension, suggesting symptoms in the language nondominant hemisphere. These findings indicate that the right temporal lobe in this case was essential for language function, which led the patient to consider other treatment options instead of focal resection of this region.

### 3.7 Case 7

A 29-year-old right-handed man developed drug-resistant focal impaired awareness seizures at 23 years of age. Long-term video EEG showed that his habitual seizures were accompanied by rhythmic beta activity in the left middle temporal region. Interictal spikes were also observed in this region. In addition, imaging studies showed an abnormal gyrus formation from the temporal to parietal lobes. Based on the overall results of these tests, the patient’s seizure focus was estimated to be on the posterolateral part of the left superior temporal gyrus, where the receptive language area is located in healthy individuals.

His left MCA was divided into three branches proximally, and the posterolateral part of the left superior temporal gyrus was fed by two branches: the M2 inf and the angular branch. We injected anesthetic into each of its two branches, but neither trial caused a decline in language function. In contrast, injection into the left M2 sup caused global aphasia. Injection into the right M1 caused transient counting errors and mild anomia, although the symptoms were much milder than those observed in the trial in the left M2 sup. Although his language function was bilaterally represented, the left temporal lobe was not considered essential for his language function.

Intracranial electrodes were implanted in the left frontotemporal areas, and ECS mapping revealed the language area in the left frontal lobe. The seizure focus in the left temporal lobe was resected without any subsequent language deficits.

### 3.8 Case 8

A 32-year-old right-handed woman developed West syndrome at the age of 1 year. She achieved remission of seizure with treatments, including adrenocorticotropic hormone therapy. However, at the age of 14 years, she developed focal impaired awareness seizures that persisted despite various drug treatments. At the age of 26 years, the patient underwent left anterior temporal lobectomy. However, the seizure frequency did not change. Long-term video-EEG monitoring revealed interictal left temporal spikes and showed that the seizures originated in the left temporal region.

ssWada revealed bilateral language representation with right-hemisphere dominance. Anesthetic injection into the right M1 segment induced motor aphasia: impairment of speech, repetition, naming, and reading aloud; however, listening and reading comprehension were preserved. Anesthetic injection into the left M1 also induced motor aphasia but to a mild degree. Her writing was relatively preserved upon anesthetic injection into the right hemisphere, with the injection into the right M2 sup inducing slight phonological agraphia and dysarthria. In contrast, anesthetic injection into the left M1 induced agraphia. Writing-related language functions appeared to be left-dominant. These findings indicated that her language functions were bilaterally distributed, oral language was predominantly on the right, and writing abilities were predominantly on the left.

Following ssWada, intracranial electrodes were placed in the left frontal and temporal lobes, and language mapping by ECS showed the language area in the left frontal lobe. The intracranial electrodes were not implanted in the right hemisphere. Considering these findings, the seizure focus in the left frontal lobe was resected. Postoperatively, mild anomia and slight repetition impairment were observed.

## 4 Discussion

We developed “ssWada,” which is a method for assessing the distribution of language areas, by refining the Wada test with advanced microcatheter technique. In this study, the novel method was applied to eight patients with drug-resistant epilepsy; of these, only three showed a typical language distribution. The Wada test is a reliable method for determining language dominance (Bauer et al., 2014, Wyllie et al., 1990). However, it cannot assess the within-hemisphere distribution of the language areas. The ssWada method is less invasive than ECS mapping and can evaluate within-hemisphere distribution of higher cognitive functions. Yamashita et al. (2021). reported a case in which fMRI hardly identified Broca's area, but ssWada successfully revealed a language area in the frontal lobe and achieved tumor resection without language impairment. They suggested that ssWada has potential utility for preoperative regional functional evaluation. It selectively suppresses regional functions and simulates the state expected to follow the resection of the majority of lesions, including subcortical areas. Two other studies (Hajek et al., 1998, Urbach et al., 2002) reported cases in which vascular-selective anesthetic infusion successfully evaluated the risk of postoperative dysfunction. Most of these patients underwent a single anesthetic injection into a single branch, and systematic language assessment was not performed. This paper reports cases in which we successfully demonstrated the intra-hemispheric distribution of different aspects of language function.

In our case series, Cases 1 and 2 showed typical language representation. In Case 2, the fMRI results were not consistent with those of the ssWada. This might be partially related to the fact that the patient was left handed. A previous study showed that language dominance prediction using fMRI is inadequate in left-handed patients or in those with atypical language lateralization (Massot-Tarrús et al., 2017, Arora et al., 2009). Studies on language dominance determined by MEG was not sufficient, especially in left-handed individuals. Similar to fMRI, language lateralization prediction by MEG in ambidextrous patients seems to be less accurate (Balaan et al., 2022).

Cases that most clearly suggested the significant role of ssWada were cases 6 and 7. In Case 6 with right temporal epilepsy, ssWada showed right hemisphere language dominance despite the previous partial resection of the right temporal lobe and long-lasting epileptic activities focused on this area. The right M2 sup showed a large perfusion area, and global aphasia occurred after anesthetic injection into this branch. In addition, the injection into the right M2 inf caused sensory aphasia. Anesthetic injection into the left M1 resulted in no language dysfunction. These findings indicate that the right temporal lobe is essential for language function, which led the patient to consider other treatment options instead of focal resection. If only a classical Wada test was performed, we would need to perform ECS to assess the language function of the right temporal lobe. In case 7, receptive language function was localized in the left frontal lobe, but not in the left temporal lobe, which is of surgical interest. If only a classical Wada test was performed, this case would have been judged unsuitable for surgery because he had left-dominant language lateralization. The ssWada opened the way for intracranial electrode implantation and focal resection of the seizure focus.

Remaining three cases (cases 4,5,8) also demonstrated atypical distribution of language areas. Case 4 showed global aphasia after anesthetic injection into the left M2 sup, which only perfused the frontal lobe. Paradoxically, she presented with well-preserved language functions despite an extensive lesion in the left temporal lobe after limbic encephalitis. Her receptive language function may have been partially compensated by her left frontal lobe. Case 5, an ambidextrous patient, showed a mirror image of the typical language distribution in the right hemisphere. Case 8, who had previously undergone a left anterior temporal lobectomy, presented with bilateral language function. Case 3 showed both speech and comprehension impairments with an anesthetic injection into the left M2 inf because his M2 inf atypically extends anteriorly to the frontal lobe. Thus, it was not considered to be an atypical distribution of brain function itself.

Our findings indicate that there is a wide variability in the distribution of language areas in patients with drug-resistant epilepsy. Despite the benefits of surgical treatment for intractable epilepsy, surgery has not been performed in patients with epileptic foci in the left hemisphere near the language area because of concerns regarding postoperative language impairment. However, ssWada provides valuable information regarding postoperative functional prognosis; it allows the simulation of postoperative functional status. This selective Wada test not only reduces the side effects of the Wada test (Fujii et al., 2011) but also provides more detailed information. Recently, fMRI combined with language tasks has been widely used for assessing language dominance, as fMRI is a noninvasive and repeatable method. While fMRI is beneficial in delineating language-related regions, it does not mean that all these regions are essential for language. ssWada is superior to fMRI in that it provides a direct indication of regional cognitive functions. Among the cases presented in this paper, all ECS-determined language distributions were consistent with the prediction by ssWada. Kemp et al. (2018). suggested that appropriate selection and complementary combinations of different methodologies will benefit patients rather than trying to determine a single best technique. The combination of the ssWada, fMRI and ECS mapping will provide sufficient information to determine the therapeutic strategies in patients with intractable epilepsy.

As a limitation, the ssWada requires highly skilled operators since catheter insertion into a cerebral artery branch requires fine control; moreover, it involves some risks, including thrombosis and hemorrhage. Previous studies report some adverse events caused by anesthetic injection into the carotid arteries (Beimer et al., 2015). However, recent advancement in devices and techniques have made this procedure much safer than before. Mikuni et al (2005) reported no permanent neurological symptoms in 58 patients who underwent the classical Wada test. Fujii et al. (2011) reported no irreversible symptoms on the MCA-selective Wada test in 17 patients. The reversible adverse effects were even lower than those reported by Mikuni et al. (2005). No irreversible complications related to the test procedures were observed in our 30 patients. Furthermore, the ssWada test is less invasive than the ECS which requires craniotomy. The noninvasive fMRI is superior to the Wada test in that it scans the entire brain using a standardized procedure and reveals the distribution of specific language functions. One advantage of ssWada is that it can directly suppress the function of the target area, which plays a role in certain suitable situations. In addition, ssWada can only segment the area to be assessed along the edges of vascular innervation. These limitations can be resolved by combining them with other tests such as fMRI or MEG.

## 5 Conclusions

ssWada is a novel functional brain localization test that can predict postsurgical functional prognosis with minor invasiveness. This test procedure could help determine whether the affected individuals would benefit from neurosurgery.

## Funding

This work was supported by Health Labor Sciences Research [grant nos. 20GB1002 and 20GC1008], Grant-in-Aid for Transformative Research Areas [grant no. 20H05956], and Grant-in-Aid for Scientific Research (B) [grant no. 21H03779] to KS. The funding sources had no involvement in the study design, collection, analysis, and interpretation of data, in the writing of the report, or in the decision to submit the article for publication.

## CRediT authorship contribution statement

**Kazuo Kakinuma:** Methodology, Investigation, Writing – original draft, Visualization. **Shin-ichiro Osawa:** Conceptualization, Methodology, Investigation, Resources, Writing – original draft, Writing – review & editing, Visualization. **Hiroaki Hosokawa:** Validation, Investigation, Data curation, Visualization, Writing – review & editing. **Marie Oyafuso:** Resources, Data curation, Writing – review & editing. **Shoko Ota:** Resources, Data curation, Writing – review & editing. **Erena Kobayashi:** Resources, Data curation, Writing – review & editing. **Nobuko Kawakami:** Resources, Data curation, Writing – review & editing. **Takafumi Sato:** Resources, Data curation, Writing – review & editing. **Mika Sakamoto:** Resources, Data curation, Writing – review & editing. **Kazushi Ukishiro:** Methodology, Investigation, Writing – review & editing. **Kazutaka Jin:** Resources, Writing – review & editing. **Makoto Ishida:** Methodology, Validation, Investigation, Data curation, Writing – original draft. **Kuniyasu Niizuma:** Resources, Writing – review & editing. **Teiji Tominaga:** Conceptualization, Writing – review & editing, Project administration. **Nobukazu Nakasato:** Resources, Writing – review & editing, Project administration. **Kyoko Suzuki:** Conceptualization, Methodology, Writing – review & editing, Supervision, Project administration, Funding acquisition.

## Ethical statement

This study was approved by the ethics committee of Tohoku University Graduate School of Medicine (2020–1–083) and conducted in accordance with the 1964 Declaration of Helsinki and its later amendments. We obtained written informed consent for the publication of patient information and images.

## Conflicts of Interest

None.
